# Unusual presentation of intraductal papilloma on the nipple: A case report

**DOI:** 10.1016/j.ijscr.2024.109483

**Published:** 2024-03-08

**Authors:** Noura Abdul Rahman, Ibrahim Arnaout, Mariam Krimsti, Amira Mardini, Kanan Rahme, Silva Ishkhanian

**Affiliations:** a Faculty of Medicine, University of Aleppo, Aleppo, Syrian Arab Republic; b Department of Dermatology and Venereology, Aleppo University Hospital, University of Aleppo, Aleppo, Syrian Arab Republic; c Department of Pathology, The Specialized Center of Dermatology, Ministry of Health, Aleppo, Syrian Arab Republic

**Keywords:** Intraductal papilloma, Nipple, Breast, Benign breast lesions, Breast neoplasms, Case report

## Abstract

**Introduction and significance:**

Intraductal papilloma (IDP) is a benign breast lesion characterized by a small, delicate wart-like growth found within the milk ducts. Typically located centrally behind the nipple, IDP often presents with a serous, serosanguinous, or bloody discharge from the nipple, making it a common cause of abnormal nipple discharge. Differential diagnosis is crucial as it can be mistaken for other conditions such as pigmented Paget's disease and pigmented basal cell carcinoma.

**Case presentation:**

This case study depicts a 35-year-old female with a painless, pigmented mass on the nipple of her right breast that had been present for four months. Physical examination revealed a well-defined blue nodule measuring 13 × 8 mm. Although mammography and ultrasound did not detect any abnormalities in the breast tissue, a biopsy confirmed the diagnosis of intraductal papilloma. The lesion was surgically excised under local anesthesia without complications.

**Clinical discussion:**

In this case, IDP presented as a blue nodule near the nipple. Despite the patient's young age and unique presentation, the diagnosis of intraductal papilloma was made based on the identified risk factors for breast tumors. Differential diagnoses considered included mammary Paget disease, nipple duct adenoma, and erosive adenomatosis of the nipple.

**Conclusion:**

This case report underscores the uncommon occurrence of IDP manifesting on the nipple. A thorough evaluation incorporating medical history, physical examination, imaging studies, and cytological analysis is essential for an accurate diagnosis and to exclude malignancy. Surgical excision was successful in removing the lesion.

## Highlights

• Intraductal papilloma (IDP) of the breast is a benign lesion characterized by a small, fragile, wart-like mass located within the lumen of lactiferous ducts.
• Differential diagnosis is crucial as it can be mistaken for other conditions such as pigmented Paget’s disease and pigmented basal cell carcinoma.
• We present a case study of a 35-year-old woman who presented with a painless, pigmented mass on the nipple of her right breast for four months.
• Rare IDP on nipple highlighted. Comprehensive evaluation crucial for accurate diagnosis. Surgical excision recommended for treatment.

## 1 Introduction

An intraductal papilloma (IDP) is a non-malignant, wart-like mass that is small, delicate, and located within the lumen of the lactiferous ducts. It is composed of fibrovascular cores that branch out and are surrounded by epithelial and myoepithelial cells. The majority of cases of intraductal papillomas are asymptomatic. There is significant variation in the age range of individuals affected by tumors, with a particular clustering of cases seen in individuals between the ages of 30 and 50 years [1].

Solitary intraductal papillomas are typically found centrally posterior to the nipple, affecting the central duct, while multiple intraductal papillomas are more commonly located peripherally in any quadrant of the breast, affecting the peripheral ducts [2]. It is very rare case where the mass was on the nipple.

Our work has been reported in line with the SCARE 2023 criteria [3].

## 2 Case presentation

A 35-year-old woman presented to the dermatology clinic with a painless and pigmented mass on her right nipple, which had been slowly increasing in size over four months. She also experienced itching and serous nipple discharge. She has no significant medical history, does not take oral contraceptive pills, She is unmarried and has never been pregnant or given birth, but has a family history of breast cancer.

**Fig. 1:**
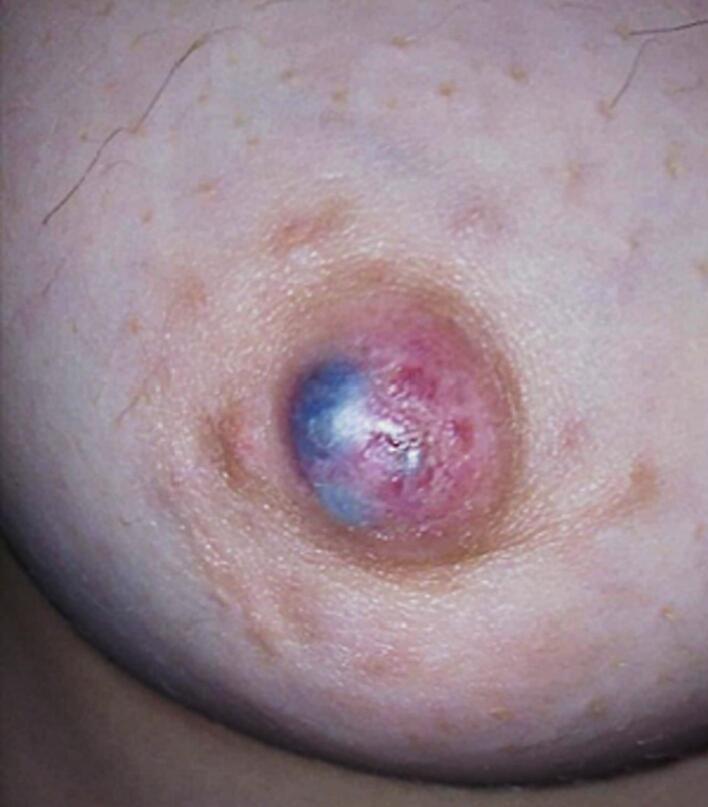
Showed a blue nodule, measuring 13 × 8 mm.

**Fig. 2:**
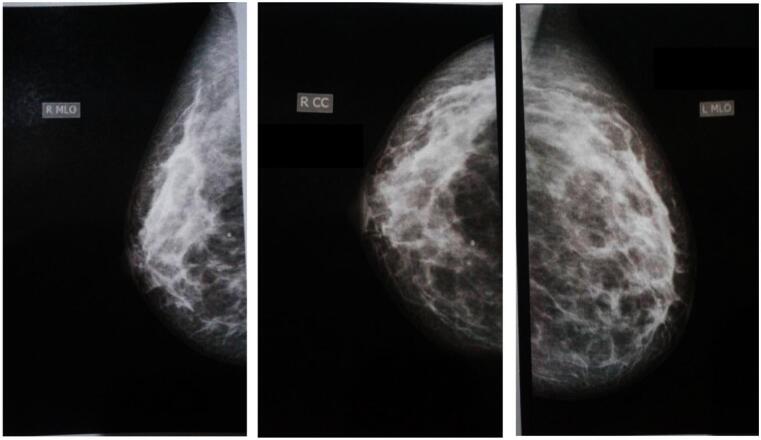
Mammography showed normal breast tissue without any masses or micro calcifications.

**Fig. 3:**
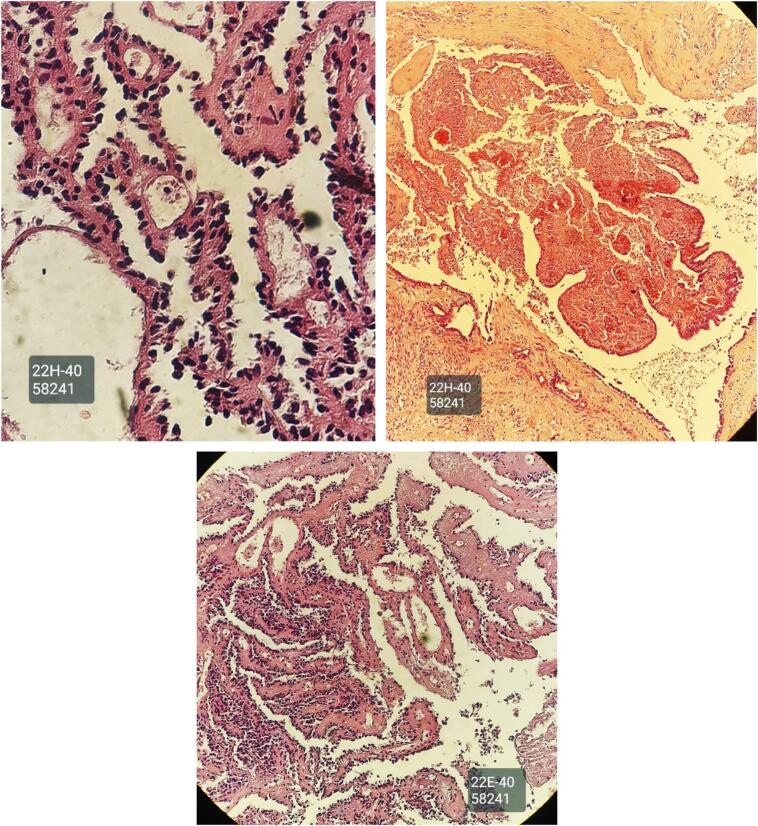
Showed part of cystic space in subcutis of breast with nearby mammary gland ducts. Lined by tall epithelial cells with myoepithelial cells, papillary projections with hyalinized fibrovascular intraluminal dissociated epithelial cells.

Incisional biopsy revealed a blue solitary well defined nodule, measuring 13 × 8 mm (Fig. 1), the skin overlying the lesion is intact. Axillary lymph nodes were not palpable. Ultrasound showed no abnormalities. Mammography showed normal breast tissue without any masses or microcalcification (Fig. 2). Histologically, sections showed part of cystic space in subcutis of breast with nearby mammary gland ducts, lined by tall epithelial cells with myoepithelial cells, papillary projections with hyalinized fibrovascular intraluminal dissociated epithelial cells. Focal sclerosing fibrosis embedding tiny ductal structures. No cellular atypia or suspicious mitotic activity (Fig. 3).

The mass was successfully removed with no complications, and the patient showed no signs of recurrence at the 6-month follow-up.

## 3 Discussion

Benign proliferative growths known as intraductal papillomas (IDP) originate from the mammary ducts. The highest incidence of intraductal papillomas occurs in women aged 30 to 50 years.

In terms of their location, IDPs can be classified as central or peripheral.

Central intraductal papillomas are generally single masses located in larger subareolar ducts and commonly associated with spontaneous nipple discharge. In contrast, peripheral intraductal papillomas are frequently multiple lesions originating in smaller ducts and seldom lead to nipple discharge [[5], [6], [7], [8]].

In this case, the intraductal papilloma manifested as a painless blue nodule on the nipple. Ultrasound imaging can show intraductal papillomas as either well-defined solid nodules or nodules attached to the duct wall within an enlarged duct [2].

In mammography, small papillomas can be challenging to identify, especially in the retroareolar areas where breast tissue density is high and compression is limited. However, larger lesions may appear as well-defined round or oval masses. Additionally, approximately a quarter of solitary papillomas may show benign calcifications on mammograms [2]. The ultrasound and mammography results in this case did not reveal any significant findings. In an incisional biopsy, a fibrovascular core typically surrounded by both epithelial and myoepithelial cells is observed. In addition to these characteristic features, intraductal papilloma may present with various alterations, including sclerosis, hyperplasia of epithelial or myoepithelial cells, atypical proliferation, and metaplasia such as squamous or apocrine changes [9].

Histopathological examination of the sections from this case revealed the presence of cystic spaces within mammary ducts, lined by tall epithelial cells accompanied by myoepithelial cells. Additionally, papillary projections with fibrovascular structures within the luminal space and focal sclerosing fibrosis were observed. The patient underwent pathological assessment with a range of potential diagnoses including mammary Paget's disease, nipple duct adenoma, erosive adenomatosis of the nipple, benign Toker cell hyperplasia, malignant melanoma in situ, basal cell carcinoma, schistosomiasis, factitial dermatitis, Bowen's disease, drug-induced eruption, and nodular localized cutaneous amyloidosis. To establish a definitive diagnosis, a thorough breast triple assessment was performed [4,10].

The development of intraductal papilloma is associated with risk factors that predispose individuals to breast tumors, including the use of contraceptives, hormone replacement therapy, prolonged estrogen exposure over a lifetime, and a family history of breast cancer [11].

The patient had a notable family history of breast cancer. The main approach to treatment involves surgical intervention with the goal of completely removing the tumor [12].

## 4 Conclusion

In this case study, we describe an unusual presentation of intraductal papilloma (IDP) affecting the nipple, which is an exceptionally rare occurrence. To confirm the diagnosis and exclude cancerous conditions, we recommend a thorough assessment involving a detailed medical history, physical examination, imaging tests, and cytological analysis.

## Abbreviation

IDP: Intraductal papilloma

## Consent

A written informed consent was obtained from the patient for publication of this case report and accompanying images. A copy of the written consent is available for review by the Editor-in-Chief of this journal on request.

## Provenance and peer review

Not commissioned, externally peer-reviewed.

## Ethical approval

The Ethics Committee in our institution approved the publication of this report.

## Funding

None.

## Author contribution

Investigation and Writing – original draft and editing: Noura Abdul Rahman, Amira Mardini.

Investigation and Writing – original draft: Kanan Rahme.

Supervision: Silva Ishkhanian, Mariam Krimsti.

Writing – review and editing and original draft: Ibrahim Arnaout.

## Guarantor

Ibrahim Arnaout.

## Research registration number

Not applicable in our case.

## Conflict of interest statement

All authors declared no conflict of interest.

## Data Availability

The corresponding author can provide the supporting data for the findings of this study upon reasonable request.
